# Shear Bond Strength of Three Orthodontic Bonding Systems on Enamel and Restorative Materials

**DOI:** 10.1155/2016/6307107

**Published:** 2016-09-21

**Authors:** Andreas Hellak, Jennifer Ebeling, Michael Schauseil, Steffen Stein, Matthias Roggendorf, Heike Korbmacher-Steiner

**Affiliations:** ^1^Department of Orthodontics, Giessen and Marburg University Hospital, Marburg Campus, Marburg, Germany; ^2^Department of Operative Dentistry and Endodontology, Giessen and Marburg University Hospital, Marburg Campus, Georg-Voigt-Strasse 3, 35039 Marburg, Germany

## Abstract

*Objective.* The aim of this in vitro study was to determine the shear bond strength (SBS) and adhesive remnant index (ARI) score of two self-etching no-mix adhesives (iBond*™* and Scotchbond*™*) on different prosthetic surfaces and enamel, in comparison with the commonly used total etch system Transbond XT*™*.* Materials and Methods*. A total of 270 surfaces (1 enamel and 8 restorative surfaces, *n* = 30) were randomly divided into three adhesive groups. In group 1 (control) brackets were bonded with Transbond XT primer. In the experimental groups iBond adhesive (group 2) and Scotchbond Universal adhesive (group 3) were used. The SBS was measured using a Zwicki 1120*™* testing machine. The ARI and SBS were compared statistically using the Kruskal–Wallis test (*P* ≤ 0.05).* Results*. Significant differences in SBS and ARI were found between the control group and experimental groups.* Conclusions*. Transbond XT showed the highest SBS on human enamel. Scotchbond Universal on average provides the best bonding on all other types of surface (metal, composite, and porcelain), with no need for additional primers. It might therefore be helpful for simplifying bonding in orthodontic procedures on restorative materials in patients. If metal brackets have to be bonded to a metal surface, the use of a dual-curing resin is recommended.

## 1. Introduction

Increasing numbers of adults have been receiving orthodontic treatment in recent years. In this situation, bonding of fixed orthodontic appliances or orthodontic attachments often has to be conducted on different prosthetic surfaces, such as crowns or cavity fillings made of metal, ceramic, or composite [1]. For an efficient workflow, it is important to establish treatment procedures that are as effective as possible, time-saving, and not subject to error. One-step adhesives were developed in the prosthetic area of dentistry. The use of these materials could be helpful in reducing the cost and effort involved in equipment in orthodontics, as a result of requiring fewer substances to achieve adequate bonding strength. In these adhesives, special primers are used to establish a durable bond following pretreatment of the bonding surface. A specific advantage of the one-step adhesives iBond and Scotchbond Universal is the fact that they contain the monomer MDP, which bonds to other materials than enamel, such as metal and ceramic surfaces [2]. When Scotchbond Universal is used, no other preliminary treatments are necessary apart from macroscopic roughening and cleaning. When iBond is used on silicate ceramic surfaces, an additional ceramic primer is needed. One-step adhesives might be particularly helpful for reducing material costs in orthodontics, with less chair-side time and the ability to avoid hydrofluoric acid.

Bonding between the adhesive and the dental enamel or prosthetic surface is decisive for treatment with a multibracket appliance. According to Brantley and Eliades, the bond strength values for conventional adhesive systems on enamel lie between 8 and 30 MPa [3]. The bond has to withstand the forces that occur in the moist oral environment and at the end of the treatment must be capable of being removed without residue and without causing damage to the enamel or prosthetic crowns, such as cracks or chipping [4]. Although shear bond strengths (SBS) in the range of 4–10 MPa have been required for bonding to enamel [5], no recommendations are currently available for bonding to different restorative materials. From the clinical point of view, the SBS on restorative materials should be at least as high as on enamel, in order to prevent high rates of bracket loss.

The classic bond to enamel is created using the acid etching technique. Preliminary treatment of the enamel with 37% phosphoric acid leads to micromechanical retention on the enamel surface [6]. Using an adhesive makes it possible to establish a bond from the enamel to the composite.

Self-etching adhesive systems were introduced as an alternative to the conventional adhesive technique. These systems simplified the technique, as etching and application of a bonding agent were now combined into a single step. Bonding to prosthetic surfaces is also simplified, since no additional primers are usually needed.

The advantages and disadvantages of these one-step adhesives have been debated in detail in the literature ever since they were first introduced. Potential advantages include reducing the time required for procedures and possibly minimizing potential errors in application [7]. In addition, self-etching adhesives appear to have advantages for use in a moist environment, due to the aqueous components contained in the primer [8].

Adequate bonding is decisive for complication-free treatment with a multibracket appliance, even on prosthetic surfaces. The aim of the present study was therefore to investigate the shear bond strength (SBS) of two one-step adhesive systems (iBond and Scotchbond Universal) in comparison with the conventional enamel etching system (Transbond XT primer) on prosthetic surfaces. Human incisors served as controls.

## 2. Materials and Methods

**Figure 1:**
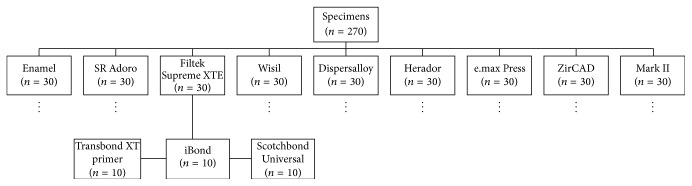
Distribution of the specimens (*n* = 270) across the different surface groups (*n* = 30) and adhesive subgroups (*n* = 10) used.

Bonding was conducted on 270 surfaces, 30 human incisors, and 240 prosthetic surfaces (Figure 1). All specimens were randomly divided into nine groups (with human enamel as the control and eight restorative surfaces as the testing groups, *n* = 30). In each group, all of the surfaces were divided into three subgroups with different adhesives (*n* = 10). The shearing tests were carried out on the basis of the DIN 13390-1 and DIN 13990-2 standards [10]. There are numerous test parameters that can influence bonding values. To ensure good comparison, all of the parameters were standardized in the present study, except for the adhesive type. Bonding to the enamel was used as the control group. Human dental enamel is the most appropriate material for testing bond strength on teeth [11]. The teeth used were extracted for general dental reasons and were obtained from dental and orthodontic practices. In relation to ethical guidelines, this represents residual biological material. The enamel surfaces were free of caries, had not been subjected to any dental treatment, and showed no enamel fractures. According to the DIN 13390-1 and DIN 13990-2 standards all extracted teeth were kept in a 0.5% tosylchloramide solution at room temperature. The storage period up to the time of testing was less than 6 months. The other 240 specimens all consisted of restorative materials used in prosthetic and conservative dentistry and were also included as bonding substrates. The specimens had a minimum size of 8 × 6 × 1 mm.

SR Adoro*™* Deep Dentin A2 (Ivoclar Vivadent, Schaan, Liechtenstein) was used as a composite, and the bulk fill composite Filtek*™* Supreme XTE (3M ESPE Dental Products, St. Paul, Minnesota, USA) was used as a composite resin.

In the alloy group, Herador*™* MP (Heraeus Kulzer, Hanau, Germany) was analyzed for the gold alloy group; Wisil*™* (Elephant Dental BV, Hoorn, Netherlands) for chrome cobalt alloys; and Dispersalloy*™* (Dentsply, Milford, Delaware, USA) for the amalgam group.

Three materials were also used as ceramics: for glass-ceramic veneering, IPS e.max*™* Press (Ivoclar Vivadent, Schaan, Liechtenstein) was used; IPS e.max ZirCAD for inLab*™* (Ivoclar Vivadent, Schaan, Liechtenstein) was used as high-strength zirconia; and VITAblocs*™* Mark II, C2 I14 for CEREC*™*/inLab (VITA Zahnfabrik, Bad Säckingen, Germany) was used as a monochromatic feldspar ceramic.

A total of 270 samples were thus available for debonding. All of the materials were used in accordance with the manufacturers' instructions.

### 2.1. Sample Preparation

The roots were cut from the teeth using a diamond saw. The enamel surfaces being tested were at least twice the size of the adhesive surface of the brackets used.

The specimens of SR Adoro Deep Dentin A2 and Filtek Supreme XTE were created by layering the composite into a mold of addition-type silicone (8 × 8 × 2 mm). The light curing with the Elipar*™* FreeLight 2 LED lamp in 400–515 nm wavelength range (3M ESPE, Seefeld, Germany) occurs to the manufacturer's instructions (time of light curing: Filtek Supreme XTE 10 s and SR Adoro 20 s). No polishing was done.

The Herador MP gold alloy was available from the manufacturer in the form of plates (8 × 8 × 1 mm) not requiring further processing. The Dispersalloy samples were stuffed into molds in a silicone form and polished to a high gloss in the laboratory after 1 day.

The chrome cobalt alloy Wisil was provided in ingots and required laboratory processing. First of all, wax probes were made in the silicone form. The probes were then placed in an embedding compound, and the Wisil probes were made in accordance with the manufacturer's instructions. After the probes had been cleaned of the embedding compound, no polishing was done.

The IPS e.max Press glass-ceramic material was also provided in ingots, so that laboratory processing was also needed, as in the Wisil group.

The zirconia IPS e.max ZirCAD for inLab samples were cut into probes 8 × 8 × 2 mm from the blocks using a diamond saw (HORICO DENTAL Hopf, Ringleb & Co. GmbH & Cie., Berlin, Germany). In accordance with the manufacturer's instruction all probes needed a laboratory process and were compacted in a sintering furnace. Consecutively there was no polishing done.

The monochromatic feldspar ceramic Mark II was also supplied in the form of blanks, which were cut in a CEREC*™* machine.

All of the probes were roughened with 50 *μ*m aluminum oxide particles using an intraoral sandblaster (MicroEtcher; Danville Materials, San Ramon, California, USA) applied from a distance of approximately 50 mm for 5 s, followed by rinsing with a water spray for 10 s and drying with oil-free compressed air. All of the surfaces of the restorative materials were degreased with alcohol.

The clinical crowns and prosthetic specimens were embedded in Palavit G*™* (Heraeus Kulzer, Wehrheim, Germany) as required by DIN 13990-2 [10]. The surfaces were oriented with their vestibular surfaces parallel to the upper end of the test tube.

The specimens were randomly divided by an external operator into three subgroups (*n* = 10) with different bonding adhesive systems:

- Transbond XT primer (3M Unitek, Monrovia, California, USA);
- iBond (Heraeus Kulzer, Hanau, Germany);
- Scotchbond Universal (3M Unitek, Monrovia, California, USA).

Table 1 lists specific information about the components of the adhesives investigated.

### 2.2. Bonding Procedure

All of the specimens were polished with Zircate*™* Prophy Paste (Dentsply DeTrey, Constance, Germany), rinsed with water, and air dried. For light polymerization, only the Elipar FreeLight 2 LED lamp (3M ESPE, Seefeld, Germany; light irradiance: 1200 mW/cm^2^, curing mode: standard, light guide: max⁡*Ø* 13 mm) was used. Light curing was done parallel to the surface at a minimum distance in the 400–515 nm wavelength range, which meets the DIN 13900-2 standard for the light source.

- In group 1 (the adhesive control group), the conventional acid etching technique was conducted for enamel. The dental enamel surfaces were conditioned with 37% phosphoric acid for 20 s and then rinsed and air dried. In the composite group (SR Adoro/Filtek Supreme XTE), a plastic conditioner (Reliance Orthodontic Products, Inc., Itasca, Illinois, USA) was applied. In the base metal group (Wisil/Dispersalloy/Herador), a metal primer (Reliance Orthodontic Products) was used, and in the ceramics group (e.max Press/ZirCAD*™*/Mark II*™*), a porcelain conditioner (Reliance Orthodontic Products) was used. The Transbond XT Primer*™* was applied afterwards using a foam pellet, thinly dispersed with air. All of the samples were light cured with the Elipar FreeLight 2 LED lamp for 10 s parallel to the surface at a minimum distance.
- In the second group, the self-etching and light curing adhesive iBond (Heraeus Kulzer, Hanau, Germany) was applied to the unconditioned enamel/prosthetic surface. It was applied to the dry enamel/prosthetic surface and rubbed in for 20 s with a single-use applicator. The liquid was then subjected to a gentle airstream for 5 s and light cured in the same way. An additional ceramic primer (Heraeus Kulzer, Hanau, Germany) was needed for 20 seconds on only two ceramic surfaces (e.max Press/Mark II).
- In the last group, the one-step adhesive Scotchbond Universal (3M Unitek, Monrovia, California, USA) was used. Following manual activation of the adhesive in the blister pack, it was applied to the unconditioned enamel/prosthetic surface, rubbed in with the single-use applicator for 20 s, and then air dried and also light cured for 10 s in the same way. No additional primers were needed.

After the use of the different adhesives on the different surfaces, the adhesive paste Transbond XT Light Cure Adhesive (3M Unitek, Monrovia, California, USA) was applied to the bracket base. To allow better comparability, only Discovery*™* upper incisor (21) steel brackets (Dentaurum, Ispringen, Germany) were used in this study. The average contact area on the bracket base was 10.95 mm^2^. Curing was then carried out again for 20 s (10 s mesial and 10 s distal) with the same light source.

Before polymerization, the brackets were applied at a pressure of 3 N with the help of a Correx*™* gauge (Haag-Streit, Berne, Switzerland), following the procedure described by Bishara et al. [12]. All of the test pieces were prepared by one person (J. E.) on 1 day. Before the shear bond testing, the specimens were stored in deionized water at 37°C for 24 ± 4 h.

**Figure 2:**
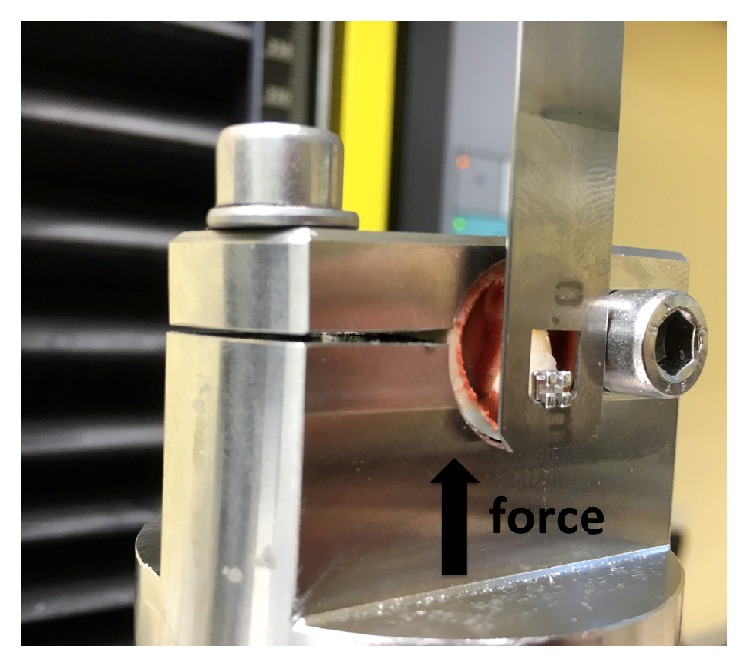
The Zwicki hydraulic testing machine with a specimen in place from lateral view.

The shear bond testing was carried out with a standardized, computer-controlled hydraulic testing machine, the Zwicki*™* 1120.25 (Zwick Ltd., Ulm, Germany) (Figure 2). The velocity of the force introduced was 1 mm/min, and the shearing force was measured in newtons (N). The clamping yoke had a square opening of 6 mm in diameter and 0.5 mm in thickness. The residual adhesive left on the base of the bracket and on the tooth surface after shearing-off was assessed using the adhesive remnant index (ARI) [13]. This allows bonding failure to be assessed (adhesive rupture versus cohesive rupture). The rupture surfaces were examined under a Leica*™* M420 microscope (Leitz, Wetzlar, Germany) at tenfold magnification.

- An ARI of 0 corresponds to 0% adhesive on the tooth and 100% adhesive on the bracket.
- An ARI of 1 corresponds to less than 50% of the adhesive on the tooth and more than 50% of it on the bracket.
- An ARI of 2 corresponds to more than 50% of the adhesive on the tooth and less than 50% of it on the bracket.
- An ARI of 3 corresponds to 100% of the adhesive on the tooth and 0% on the bracket.
- An ARI of 4 means a surface fracture.

For purposes of better comparability, the resulting forces were converted into MPa in accordance with the following formula:

(1) $$\begin{array}{c} R\;\;\left(\;N/{mm}^{2}\;\right)=\frac{F\;\left(N\right)}{A\;\left({mm}^{2}\right)}, \end{array}$$

where *R* is cohesive bond strength, *F* is force, and *A* is the cross-sectional surface of the adhesive test piece. The relative value calculated allows comparisons with other studies.

Statistical analysis was carried out using IBM SPSS Statistics*™* for Macintosh, version 21.0 (IBM Corporation, Armonk, New York, USA). Normal distribution was tested using the Shapiro–Wilk test. Testing with the Shapiro–Wilk test showed that the values were not normally distributed. Nonparametric tests were therefore used. Statistical differences were analyzed using the Kruskal–Wallis test. The Kaplan–Meier survival curve and log rank test were used to test similarity. The significance level for all of the analysis procedures was set at *P* ≤ 0.05.

## 3. Results

The three adhesives showed different bond strengths on enamel and prosthetic surfaces (Table 2). In descriptive comparisons, Table 2 shows that the Transbond XT adhesive system had the highest (mean) values for shear bond strength (15.51 MPa) on enamel. The highest mean values on all surfaces were obtained with Scotchbond Universal on the Filtek Supreme XTE surface (16.61 MPa) and were average on all other surfaces. Most of the SBSs were higher than the SBS on enamel required by Reynolds [9]. The lowest means were achieved on e.max Press with iBond adhesive (3.44 MPa) and in general on metal surfaces (especially on Herador) with all of the different adhesives. These SBSs were sometimes lower than required for clinical use [9].

**Figure 3:**
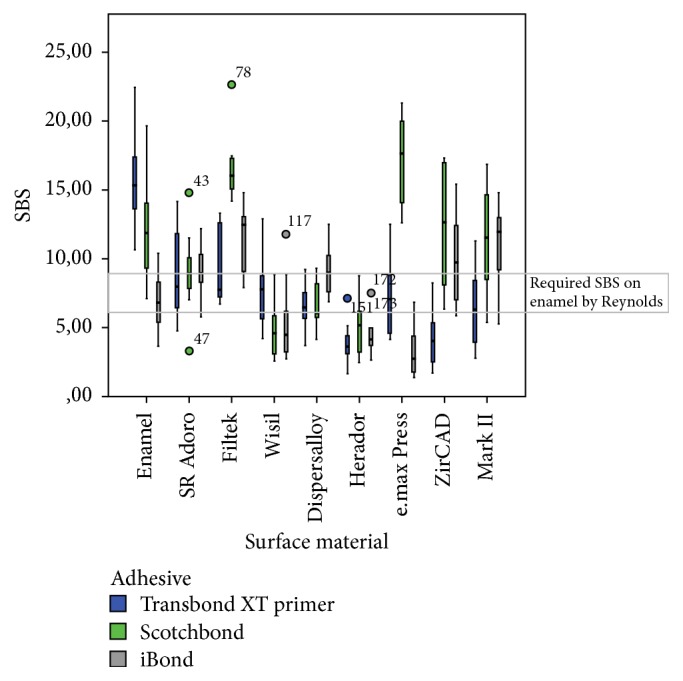
Distribution of shear bond strength (SBS) in MPa in the different adhesives and surfaces used (*n* = 270). All three adhesives showed different bond strengths, mostly higher and sometimes lower than the values required by Reynolds on enamel [9]. The circles indicate outliers.

The distributions of shear bond strengths in the various adhesive systems are summed up graphically as a box plot diagram in Figure 3 (circles indicate outliers). The Kruskal–Wallis test showed that there were highly significant to nonsignificant differences between the adhesives in the surface groups (Table 3). Testing for similarity using the Kaplan–Meier survival curve and log rank test also showed that there were significant differences in the survival distributions. The Kaplan–Meier curves showed that, on some surfaces, most of the adhesives showed a lower cumulative survival than the minimum required by Reynolds [9].

**Figure 4:**
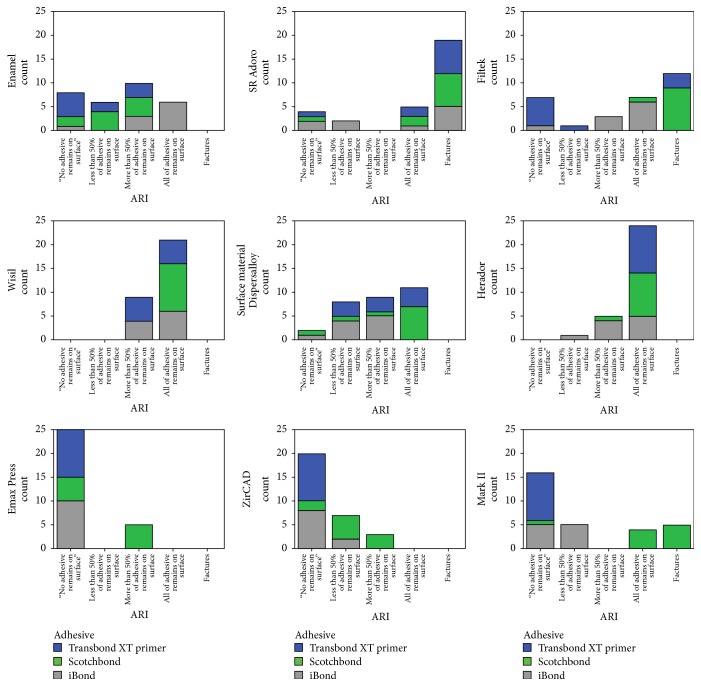
Distribution of the adhesive remnant index (ARI) scores for the three different adhesives used in this study (Transbond XT = blue, iBond = grey, and Scotchbond = green; *n* = 270). An ARI of 0 corresponds to 0% adhesive on the tooth and 100% adhesive on the bracket. An ARI of 1 corresponds to less than 50% of the adhesive on the tooth and more than 50% of it on the bracket. An ARI of 2 corresponds to more than 50% of the adhesive on the tooth and less than 50% of it on the bracket, and an ARI of 3 corresponds to 100% of the adhesive on the tooth and 0% on the bracket. An ARI of 4 corresponds to surface fractures.

**Figure 5:**
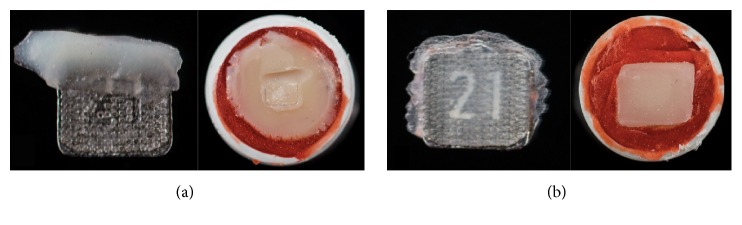
Typical examples of adhesive remnant index (ARI) scores of the bracket base and surface. (a) ARI score 4, with fractures in the group bracket base and surface; (b) ARI score 0, with 0% of the adhesive on the surface and 100% adhesive on the bracket.

The quality of the bonding failure mode was examined and evaluated under a microscope at tenfold magnification. Statistical analysis of the distribution of the ARI scores again showed that they were not normally distributed. The Kruskal–Wallis test showed that there were highly significant to nonsignificant differences (Table 3).  Figure 4 shows the distribution of the ARI scores for the different primers, and Figures 5(a) and 5(b) provide typical examples illustrating ARI scores.

## 4. Discussion

The three adhesive systems investigated in this study showed adhesive strength values on enamel that satisfied or were greater than the minimum required by Reynolds (5.9–7.8 MPa) for the clinical use of brackets [9]. Comparisons in the enamel group showed highly significant differences between the three adhesives with regard to shear bond strength. iBond in particular showed a lower SBS. The view held by several authors that only a weaker bond can be expected is thus confirmed [14].

The Transbond XT primer can be regarded as one of the standard adhesive systems in orthodontics. It has been the subject of many studies examining its adhesive strength [9, 12, 15–17]. In the present study, a mean value was measured for the Transbond XT primer that was comparable to that reported in other studies for the bracket-adhesive bond [18, 19]. From the authors' point of view, Transbond XT primer with a conventional acid etching technique can still be regarded as the gold standard for bonding brackets on enamel, except in special clinical situations, as mentioned below.

The second bonding system, iBond, also showed SBS values similar to those reported in the literature [20]. In comparison with the last adhesive, it needs to be pointed out that Scotchbond*™* and Scotchbond Multipurpose Plus*™* are not the same as Scotchbond Universal, although they sound similar. Scotchbond Universal is a further development of Adper Easy Bond*™*, which has been available since December 31, 2012.

A literature search did not identify any comparable studies using a similar study design for the Scotchbond Universal adhesive system, the third adhesive system used in the present study. A few publications about Scotchbond Universal are only concerned with the prosthetic area. Takamizawa et al. [21] reported much higher SBS values, which are not essential for bracket bonding (28.4–48.6 MPa). Comparison with the orthodontic area would be difficult, as these SBSs might lead to unwanted enamel fractures during debonding (ARI = 4).

One advantage of self-etching adhesives is that the substance can be used in a moist environment, due to the aqueous components in the self-etching primer. Hydrophilic adhesive systems are able to repel moisture from the enamel surface, so that the adhesive can penetrate the unconditioned enamel without obstruction [8]. In contrast to conventional adhesive systems, therefore, no absolute drying is required. This can have positive effects, above all when bonding brackets in the inferior and posterior teeth [22, 23], since it makes adhesion easier especially on exposed teeth, as the enamel surface being glued is quite often contaminated with saliva or even blood. As described in the literature in these clinical situations, self-conditioning adhesive systems have better bond strength values than conventional adhesive systems [24, 25]. The shallower etching pattern in self-conditioning adhesive systems leads to less dissolution of the dental enamel, resulting in reduced loss of hard tooth tissue [26]. The study by Hosein et al. [26] found that, during the process of etching the enamel with self-conditioning adhesive systems, the enamel loss was lower, at 0.03–0.74 *μ*m, than with conventional adhesive systems, at 1.11–4.57 *μ*m.

No specialized products are currently available for bonding orthodontic brackets to restorative materials. As required by Reynolds 30 years ago, the SBS should not be below the cohesive strength of enamel, in order to avoid enamel fractures during debonding [9]. If prosthetic tooth surfaces are bonded with the bracket, the debonding will not damage the enamel, but there is nevertheless a risk of inducing defects or cracks on crowns, veneers, fillings, or other types of restored surface [27]. Scotchbond Universal and iBond are described by their manufacturers as generating reliable bonds for permanent indirect restorations in prosthetics and conservative dentistry. According to the manufacturers' information, these adhesives contain the monomer MDP, which also creates an adhesive bond with composite, metal, and ceramic surfaces. In this study, the highest means for all prosthetic surfaces were obtained with Scotchbond Universal without an additional primer. The only pretreatment used was sandblasting with alumina particles. Scotchbond Universal may therefore be helpful for reducing equipment costs in orthodontics, as fewer substances need to be used to achieve similarly adequate adhesive bond strengths with different materials. In case of iBond on ceramic surfaces, only one additional ceramic primer is recommended. This might be a disadvantage, as two steps are needed for adequate bonding on glass-ceramic and monochromatic feldspar ceramic surfaces. In the case of Transbond XT, three additional primers (plastic conditioner, metal primer, and porcelain conditioner) are needed. In conclusion, one-step adhesives may be particularly helpful for reducing material costs. Another advantage might be that eliminating the need for selective etching on enamel and bonding brackets, without an additional primer on prosthetic surfaces, may reduce the risk of errors during application and may reduce the amount of chair time [28]. In some surface groups, however, SBS values lower than the minimum required by Reynolds (5.9–7.8 MPa) were found, especially in the metal groups [9]. This might lead to incomplete curing of the adhesive, as not enough light can enter the gap between the light-opaque bracket base and the restorative surface [27]. In this case, the use of a dual-curing resin has to be recommended. With regard to composite [29, 30] and ceramic [31] materials in the present study, higher mean values as well as comparable values were found.

In general, the bonding strength of the adhesive system used should only be large enough to resist the forces that arise in the orofacial region. Contrasting with this, there is the requirement that the system must be easy to remove without causing iatrogenic damage such as chipping and cracking of the enamel or prosthetic surface [4]. In contrast to the requirements for composite fillings in conservative dentistry, where the fillings are intended to remain in place for as long as possible, an adhesive that is used in orthodontics has to be removable at the end of the course of treatment without causing any harm to teeth or restorative material. Once the goal of the treatment has been achieved, a multibracket device must be completely removable. The results of the adhesive remnant index show an inhomogeneous distribution for the three bonding systems. At least some of the adhesives showed an ARI value of 4. In this situation, cracks or fractures on prosthetic restorations were detected. Some of these adhesives might therefore not be safe for clinical use (Figure 5(a)).

Shear bond tests are a recognized in vitro testing procedure for measuring adhesive force. To allow better comparison of the results obtained, they are converted by many authors from N/mm^2^ into MPa [32]. There are numerous testing parameters that can influence in vitro adhesiveness values—such as the type of adhesive used, the material properties of the bracket base, the way in which the test pieces are stored, the diameter of the adhesive gap, the shearing velocity of the test machine, the type and duration of light-curing, and the dental or prosthetic material used. With the exception of the adhesive type, all of the other parameters were standardized in the present study to the DIN 13390-1 and DIN 13990-2 standards. Variabilities in the interindividually differing structure of the human enamel are negligible, with a test figure of 10 as required in the test standard [10].

In general, the results of in vitro experiments are never precisely comparable with those of in vivo situations, since application-sensitive substrates and the complexity of the interactions involved are subject to error, and standardization can never succeed 100% [4]. However, the results of in vitro experiments can provide important information for in vivo situations and are of decisive value for clinical practice and everyday clinical use.

## 5. Conclusions

Within the limitations of an in vitro study, Transbond XT showed the highest SBS on human enamel and can still be regarded as the gold standard on enamel. Scotchbond Universal provides the best average bonding on all other types of surface (metal, composite, and porcelain), with no need for additional primers. It might therefore be helpful for simplifying bonding in orthodontic procedures on restorative materials. If metal brackets have to be bonded to a metal surface, the use of a dual-curing resin is recommended. Further in vivo studies will be needed in order to obtain clinical confirmation of these promising results.

## Figures and Tables

**Table 1:** Specific information about the components of the adhesives investigated. Etching: Ormco® Gel Etch phosphoric acid 5 mL (Ormco Corporation, Glendora, California, USA).

Adhesive	Pack contents and batch identifier
Transbond XT	Light cure adhesive primer, batch number 8FB/712-034
	Light cure adhesive paste, batch number 8CU

iBond	1a iBond ceramic primer, batch code 010089
	1b iBond Universal, batch code 010020

Scotchbond Universal blister	1a MDP phosphate monomer, dimethacrylate
	1b HEMA, Vitrebond copolymer, filler, ethanol, water, initiators, and silane

**Table 2:** Descriptive statistics on SBS values for the different bonds used on enamel and prosthetic surfaces.

Surface material	Adhesive		Shear bond strength
Enamel	Transbond XT primer	Mean	15.51
		*n*	10
		SD	3.49

Enamel	Scotchbond	Mean	12.09
		*n*	10
		SD	3.85

Enamel	iBond	Mean	6.96
		*n*	10
		SD	2.00

Enamel	*Total*	Mean	11.52
		*n*	30
		SD	4.73

SR Adoro	Transbond XT primer	Mean	8.82
		*n*	10
		SD	3.39

SR Adoro	Scotchbond	Mean	9.03
		*n*	10
		SD	3.02

SR Adoro	iBond	Mean	8.99
		*n*	10
		SD	1.94

SR Adoro	*Total*	Mean	8.94
		*n*	30
		SD	2.75

Filtek Supreme XTE	Transbond XT primer	Mean	9.32
		*n*	10
		SD	2.80

Filtek Supreme XTE	Scotchbond	Mean	16.61
		*n*	10
		SD	2.38

Filtek Supreme XTE	iBond	Mean	11.43
		*n*	10
		SD	2.58

Filtek Supreme XTE	*Total*	Mean	12.45
		*n*	30
		SD	4.00

Wisil	Transbond XT primer	Mean	7.62
		*n*	10
		SD	2.37

Wisil	Scotchbond	Mean	4.69
		*n*	10
		SD	1.84

Wisil	iBond	Mean	5.39
		*n*	10
		SD	2.89

Wisil	*Total*	Mean	5.90
		*n*	30
		SD	2.65

Dispersalloy	Transbond XT primer	Mean	6.62
		*n*	10
		SD	1.61

Dispersalloy	Scotchbond	Mean	6.71
		*n*	10
		SD	1.70

Dispersalloy	iBond	Mean	9.28
		*n*	10
		SD	1.92

Dispersalloy	*Total*	Mean	7.54
		*n*	30
		SD	2.10

Herador	Transbond XT primer	Mean	3.78
		*n*	10
		SD	1.50

Herador	Scotchbond	Mean	5.14
		*n*	10
		SD	1.89

Herador	iBond	Mean	4.69
		*n*	10
		SD	1.63

Herador	*Total*	Mean	4.54
		*n*	30
		SD	1.72

e.max Press	Transbond XT primer	Mean	7.07
		*n*	10
		SD	2.62

e.max Press	Scotchbond	Mean	17.20
		*n*	10
		SD	3.21

e.max Press	iBond	Mean	3.44
		*n*	10
		SD	2.05

e.max Press	*Total*	Mean	9.24
		*n*	30
		SD	6.46

ZirCAD	Transbond XT primer	Mean	4.29
		*n*	10
		SD	2.18

ZirCAD	Scotchbond	Mean	12.33
		*n*	10
		SD	4.15

ZirCAD	iBond	Mean	10.01
		*n*	10
		SD	3.08

ZirCAD	*Total*	Mean	8.88
		*n*	30
		SD	4.65

Mark II	Transbond XT primer	Mean	6.37
		*n*	10
		SD	2.72

Mark II	Scotchbond	Mean	11.16
		*n*	10
		SD	3.76

Mark II	iBond	Mean	10.85
		*n*	10
		SD	3.13

Mark II	*Total*	Mean	9.46
		*n*	30
		SD	3.83

**Table 3:** The Kruskal–Wallis test showed highly significant to nonsignificant differences in shear bond strength (SBS) and adhesive remnant index (ARI) in the three groups.

Surface material		SBS	ARI
Enamel	Chi-square test	18.512	11.686
	Df	2	2
	Asymptotic significance	0.000	0.003

SR Adoro	Chi-square test	0.281	1.636
	Df	2	2
	Asymptotic significance	0.869	0.441

Filtek Supreme XTE	Chi-square test	20.847	14.017
	Df	2	2
	Asymptotic significance	0.000	0.001

Wisil	Chi-square test	6.970	6.444
	Df	2	2
	Asymptotic significance	0.031	0.040

Dispersalloy	Chi-square test	9.223	6.691
	Df	2	2
	Asymptotic significance	0.010	0.035

Herador	Chi-square test	3.583	8.561
	Df	2	2
	Asymptotic significance	0.167	0.014

e.max Press	Chi-square test	23.086	11.600
	Df	2	2
	Asymptotic significance	0.000	0.003

ZirCAD	Chi-square test	16.423	15.612
	Df	2	2
	Asymptotic significance	0.000	0.000

Mark II	Chi-square test	9.762	19.815
	Df	2	2
	Asymptotic significance	0.008	0.000

Df: degrees of freedom (statistics).
